# Traditional and emerging strategies using hepatocytes for pancreatic regenerative medicine

**DOI:** 10.1111/1753-0407.13545

**Published:** 2024-04-10

**Authors:** Shuang Liu, YuYing Zhang, YunFei Luo, JianPing Liu

**Affiliations:** ^1^ Department of Metabolism and Endocrinology, the Second Affiliated Hospital, Jiangxi Medical College Nanchang University Nanchang China

**Keywords:** CRISPR/Cas9, epatocytes, organoids, regenerative medicine

## Abstract

Although pancreas and islet cell transplantation are the only ways to prevent the late complications of insulin‐dependent diabetes, a shortage of donors is a major obstacle to tissue and organ transplantation. Stem cell therapy is an effective treatment for diabetes and other pancreatic‐related diseases, which can be achieved by inducing their differentiation into insulin‐secreting cells. The liver is considered an ideal source of pancreatic cells due to its similar developmental origin and strong regenerative ability as the pancreas. This article reviews the traditional and emerging strategies using hepatocytes for pancreatic regenerative medicine and evaluates their advantages and challenges. Gene reprogramming and chemical reprogramming technologies are traditional strategies with potential to improve the efficiency and specificity of cell reprogramming and promote the transformation of hepatocytes into islet cells. At the same time, organoid technology, as an emerging strategy, has received extensive attention. Biomaterials provide a three‐dimensional culture microenvironment for cells, which helps improve cell survival and differentiation efficiency. In addition, clustered regularly interspaced short palindromic repeats (CRISPR)/Cas9 gene editing technology has brought new opportunities and challenges to the development of organoid technology.

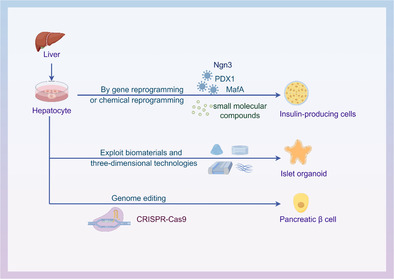

## Graphical abstract

Highlights

- At present, the traditional and emerging strategies using hepatocyte‐derived islet‐like cells for diabetic cell replacement therapy have made extensive and far‐reaching progress. Gene reprogramming and chemical reprogramming technology are traditional strategies with potential, and organoid technology as an emerging strategy has also been widely studied. Clustered regularly interspaced short palindromic repeats (CRISPR)/Cas9 gene editing also provides new opportunities and challenges for the development of organoid technology.

## 1 BACKGROUND

Diabetes mellitus is a metabolic disease characterized by chronic hyperglycemia, which has long been a global health crisis.^1^ During the onset of diabetes, the number of islet β cells is reduced, the function is impaired, and it is very difficult to recover. At present, traditional treatment methods can relieve the symptoms of patients with high blood sugar only to a certain extent and cannot fundamentally treat diabetes. Pancreas and islet cell transplantation is the only treatment to prevent the late complications of insulin‐dependent diabetes mellitus.^2^, ^3^ However, the lack of donors is the biggest obstacle to organ transplantation, and although many strategies have been developed to solve this problem, great challenges remain. Stem cell therapy is an effective way to cure diabetes and other pancreatic‐related diseases, which can be achieved by different methods of inducing insulin‐producing cells. Many studies have used key genes and signals of the pancreatic lineage to generate insulin‐producing β‐like cells from stem cells, based on reference to normal pancreatic β‐cell development patterns.^4^

**FIGURE 1:**
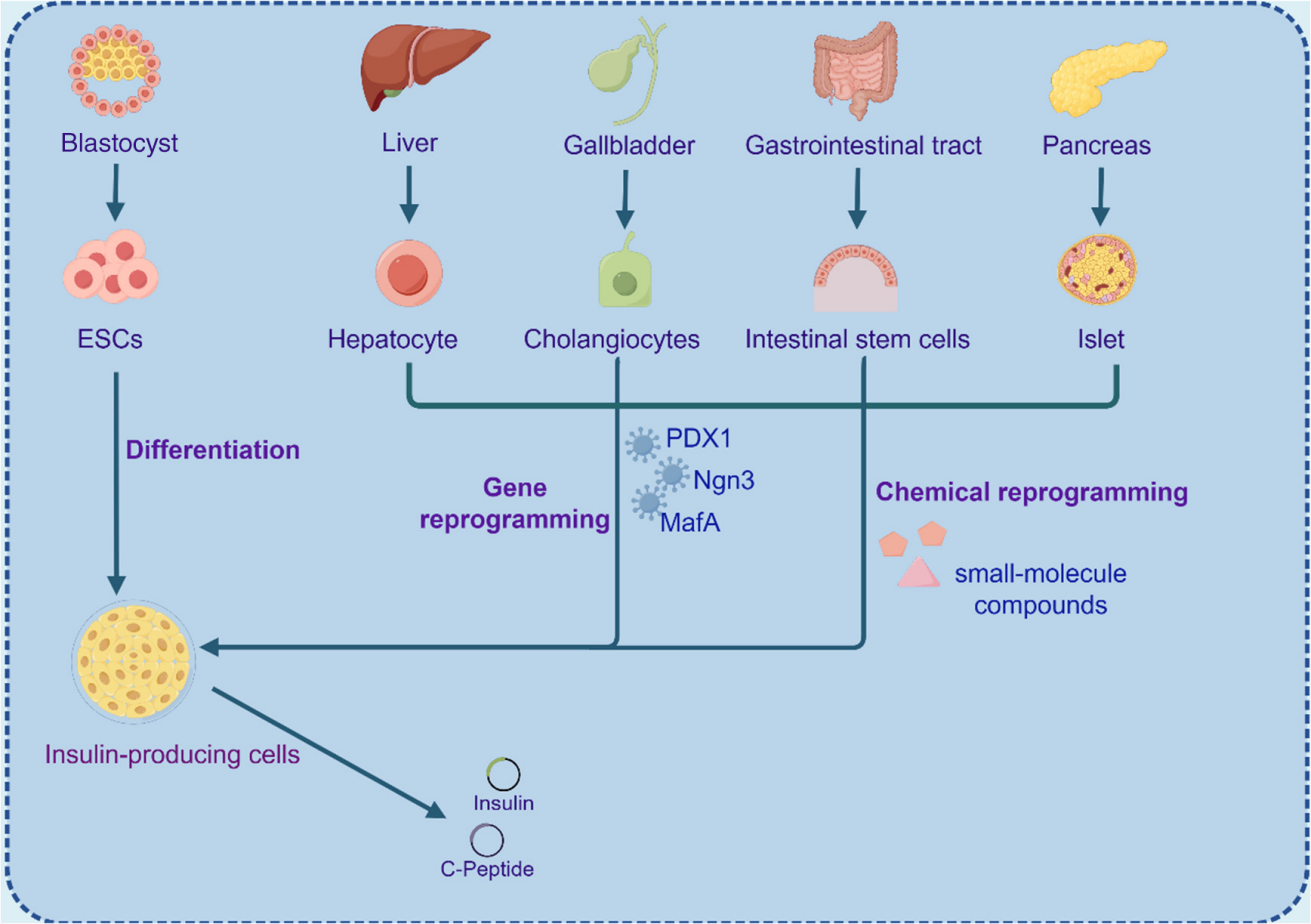
Insulin‐producing cells derived from stem cells. ESC, embryonic stem cell; MafA, macrophage activating factor bZIP transcription factor A; Ngn3, neurogenin3; PDX1, pancreatic duodenal homeobox‐1.

Stem cells can be divided into embryonic stem cells (ESCs) and adult stem cells (Figure 1). ESCs can proliferate indefinitely in vitro, but there are ethical issues and risks of tumorigenesis.^5^ Adult stem cells are distributed in various tissues and organs, with stable differentiation and high safety, and are entering clinical research, and their safety is much higher than that of ESCs. Among the many sources of adult stem cells, the liver is considered an ideal cell source for the production of pancreatic cells because of its developmental origin close to pancreatic organs, strong regenerative capacity and accessibility, and the absence of the safety risks associated with stem cells.^6^ During embryonic development, cells of the liver, bile ducts, and pancreas lineage are derived from common endoderm progenitor cells. Hepatocytes can be guided by genetic and chemical reprogramming techniques to emulate pancreatic organogenesis, progressing through developmental stages similar to the final endoderm, primitive intestinal endoderm, hind foregut, pancreatic endoderm, and endocrine precursor. This process leads to their differentiation into insulin‐secreting cells in vitro, displaying numerous functional characteristics of β cells such as the expression of transcription factors in β cells, responsiveness to elevated glucose levels, secretion of C‐peptide, and the presence of mature endocrine secretion granules.^7^

Nevertheless, the induced cells remain in single‐cell mode, and to improve this, an appropriate microenvironment with oxygen gradients, nutrients, favorable extracellular matrices, and the addition of more potent small molecule compounds or growth factors needs to be developed. Pancreatic organoid strategies of the medium can also be improved by adding the necessary factors and using biocompatible scaffolds to achieve long‐term functional capacity after transplantation.^8^, ^9^, ^10^ Organoid technology, which exhibits properties that mimic the development of human organs, enables cell therapy, and reconstructs damaged or diseased tissues, has great potential in the future.^11^, ^12^ In addition, clustered regularly interspaced short palindromic repeats (CRISPR)/Cas9 technology can also correct genetic abnormalities in vitro and reimplant healthy transgenic cells into patients. With the continuous development of the organoid culture system and its experimental development technology, it has great significance and application value for pancreatic regeneration research.

**FIGURE 2:**
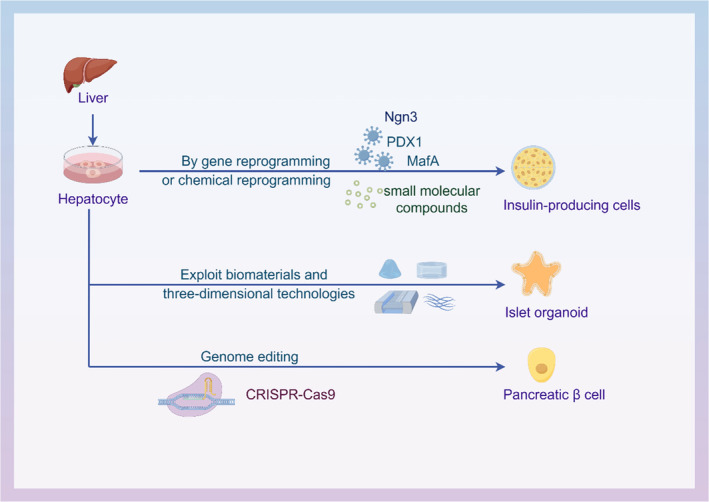
Traditional and emerging strategies of hepatocytes in pancreatic regenerative medicine. CRISPR, clustered regularly interspaced short palindromic repeats; MafA, macrophage activating factor bZIP transcription factor A; Ngn3, neurogenin3; PDX1, pancreatic duodenal homeobox‐1.

Unlike current organ transplant therapies, the generation of in vitro‐derived autologous tissue from patient cells is not affected by immune capacity and rejection, providing alternative organ replacement strategies. This review focuses on the traditional and emerging strategies using hepatocytes for pancreatic regenerative medicine, further elucidating the theoretical and practical basis of hepatocytes in pancreatic regeneration and pointing out the direction for more effective clinical treatment (Figure 2).

## 2 GENE REPROGRAMMING TECHNOLOGY OF HEPATOCYTES IN PANCREATIC REGENERATIVE MEDICINE

Gene reprogramming is a promising approach for hepatocytes in pancreatic regenerative medicine. Hepatocytes and pancreatic β cells share gene expression profiling similarities, including related transcription factors, glucose transporter 2 (*GLUT2*) and glycerol kinase (*GK*) genes. Hepatocytes can be transformed into a stem cell‐like state by intervention of transcription factors and other molecules and then further differentiate into pancreatic cells. Transcription factors play a key role in regulating gene expression. Ectopic expression of specific transcription factors can alter or transdifferentiate somatic cell fate. According to reports, cells of various origins including pancreatic exocrine cells, hepatocytes, enterocytes, gallbladder cells and stem cells can express pancreatic development‐related transcription factors (pancreatic duodenal homeobox‐1 [PDX1], neurogenin3 [Ngn3], macrophage activating factor bZIP transcription factor A [MafA], forkhead box protein A2 [FOXA2], SRY‐box transcription factor 17 [Sox17], paired box 6 [Pax6], etc.) can be transformed into insulin‐producing cells.^13^, ^14^ *PDX1* is the main regulatory gene for the development of islet endocrine cells.

### 2.1 PDX1 is key in transcription factor reprogramming strategies

*PDX1* plays a key role in early embryonic pancreas formation, directed differentiation of different endocrine lineages, and late maturation of β cell function.^15^ *PDX1* expression was detected on the 8.5th day of mouse embryonic development (E8.5) and around the fourth week of human gestation.^13^, ^16^ As early as 2000, Ferber et al demonstrated the capacity of *PDX1* to reprogram extrapancreatic tissue into a similar β cell phenotype.^17^ To study the effects of ectopic expression of *PDX1* in the liver, the researchers delivered *PDX1* recombinant adenovirus to male mice aged 11–14 weeks. The results showed that *PDX1* was mainly expressed in the liver, whereas the expression of this gene induced the expression of endogenous mouse insulin 1 (mI‐1) and mI‐2 genes in the liver. A single *PDX1* gene expression is sufficient to induce the expression and activation of endogenous insulin genes in tissues outside the pancreas. When PDX1 recombinant adenovirus was delivered to streptozocin (STZ)‐induced diabetic mice for treatment, blood glucose levels gradually decreased. This suggests that the expression of *PDX1* is sufficient to induce mature, biologically active insulin production in the liver. In islets, PDX1 works synergistically with other transcription factors to regulate the expression of insulin and other islet‐specific genes.^18^, ^19^ Transcription factors that occur naturally in the liver are ubiquitous and tissue specific and may work synergistically with ectopic PDX1 to induce and regulate insulin gene expression in this organ. In 2006, Yamada et al introduced the adenovirus‐mediated *PDX1* gene into hepatocytes cultured in vitro and detected insulin expression at the mRNA and protein levels. Hepatocyte albumin expression is downregulated during transdifferentiation, and cytokeratin‐19 (*CK19*) and alpha‐fetoprotein mRNA are upregulated. These results suggest that hepatocytes have the potential to transdifferentiate into insulin‐producing cells in vitro. Transcriptome analysis showed that fetal hepatocytes introduced into *PDX1* expressed a variety of β cell‐related genes as well as genes expressed in non‐β islet cells, such as glucagon and pancreatic polypeptide (*PP*). Although cells respond to glucose stimulation, they secrete very low levels of insulin, about 2% of normal human islets.^20^ These results suggest a large difference between hepatocytes transdifferentiated with *PDX1* alone and islet β cells.

### 2.2 PDX1 and Ngn3 combine to improve the efficiency of the transcription factor reprogramming strategy

Ngn3 encodes an essential helix‐ring‐helix transcription factor that is also considered a major regulator of pancreatic development.^21^, ^22^ At the mouse embryonic stage of 8.5 days (E8.5), *Ngn3* was first detected during the primary transition of islet development, and E11.5 expression decreased to near zero. *Ngn3* is reexpressed during the secondary transition of islet development and peaks at E15.5.^23^ Pancreatic endocrine cells disappear in mice with *Ngn3* gene deletion and die shortly after birth.^22^ In the adult mouse pancreas, a small number of Ngn3 expression‐positive cells can be detected at normal homeostasis. Based on lineage tracing, these Ngn3‐positive cells are a population of islet progenitor cells.^24^ In damaged pancreatic tissue, Ngn3 expression is upregulated, and Ngn3‐positive cells contribute to adult β cell regeneration.^25^, ^26^ When expressed ectopically in non‐endo epithelial cells, *Ngn3* can confer endocrine cell fate on these cells.^22^ *Ngn3* is necessary for pluripotent stem cell‐derived maturation of human β cells.^27^ Previous studies have shown that expression of *PDX1* in the liver via adenovirus vectors can produce some insulin‐expressing cells in the liver and alleviate symptoms of diabetes in animal models.^17^, ^28^ In a 2005 study, researchers' overexpression of PDX1, which carries a VP16 transcriptional activation domain modification, along with NeuroD or Ngn3, significantly increased insulin gene promoter activity in HepG2 cells and was more effective than wild‐type PDX1. Furthermore, PDX1/VP16 overexpression along with NeuroD or Ngn3 significantly improves glucose tolerance in STZ‐induced diabetic mice.^29^

However, the study did not determine whether those cells that expressed insulin were β‐like cells. In a 2007 study, Soonsang Yoon demonstrated that coexpression of PDX1 and Ngn3 produced a small number of β‐like cells in the liver.^30^ Coexpression of PDX1 and Ngn3 induces the expression of various islet cell‐specific genes to initiate a series of events that induce a small number of hepatocytes into β‐like cells. Chang et al showed that PDX1 and Ngn3 combined can reprogram hepatocytes into platelet‐derived growth factor receptor A (PDGFRα)‐positive β‐like cells.^31^ The addition of PDGF‐AA to glucagon‐like peptide‐1/exendin‐4‐containing medium promotes the expansion of β‐like cells, and the cells can secrete insulin in response to glucose stimulation, enabling the treatment of diabetic mice after transplantation. Compared to previous work using PDX1 alone to reprogram hepatocytes into insulin‐producing cells, the improved strategy developed based on the current work not only improves the efficiency of reprogramming hepatocytes into β‐like cells but also enhances the ability of reprogrammed cells to secrete insulin.

### 2.3 Specific combinations of Ngn3, PDX1, and MafA are central to the transcription factor reprogramming strategy

MafA is a transcription factor with a leucine zipper structure that belongs to the macrophage activating factor (MAF) family.^32^ MafA is the only specific islet β cell insulin gene‐activating transcription factor found so far, which can bind to the conserved cis‐regulatory element RIPE3b in the insulin gene promoter region as a strong transaction factor to regulate insulin expression. Islet β cell maturation and functional maintenance depends on the normal expression of the MafA protein.^33^, ^34^, ^35^

In 2008, Zhou et al identified 20 transcription factors specifically expressed in embryonic pancreatic cells out of 1100 by in situ hybridization.^22^ Of these, nine genes were highly correlated with pancreatic development and were selected for the initial reprogramming experiment. A specific combination of three transcription factors, Ngn3, PDX1, and MafA, was identified, which reprogram pancreatic exocrine cells into cells that closely resemble islet β‐cells. Induced islet β‐like cells are indistinguishable from endogenous islet β cells in size, shape, and ultrastructure. They express genes necessary for β‐cell function and can improve hyperglycemia by remodeling the local vascular system and secreting insulin.

Banga et al showed that the combination of PDX1, Ngn3, and MafA transcription factors was also able to reprogram cells in the liver into insulin‐positive cells.^36^ These cells exhibit duct‐like structures expressing epithelial‐specific marker proteins E‐Cadherin and CK19 while being positive for insulin and C‐peptide. The content of insulin released after the corresponding glucose stimulation reached 20% of the islets of normal mice. However, these cells also secrete other pancreatic hormones. The study also showed that the combination of PDX1, Ngn3, and MafA was more reliable and effective than previously used combinations, with liver cells maintaining insulin expression for longer.

## 3 CHEMICAL REPROGRAMMING TECHNOLOGY OF HEPATOCYTES IN PANCREATIC REGENERATIVE MEDICINE

The emergence of induced pluripotent stem cells (iPSCs) proves that cells can be reprogrammed through a combination of transcription factors to alter cell fate.^37^ This method has been widely used to induce transitions between other types of cells.^15^ However, the risk of insertion of exogenous DNA sequences and reactivation of exogenous genetic factors, as well as unpredictable side effects associated with ectopic gene expression, raise safety concerns for clinical applications.^38^ Although transient delivery through nonintegrated pathways of foreign genes or delivery using special particles has been reported, high cost, low delivery efficiency, and technical difficulty hinder the application of these techniques.^39^

**FIGURE 3:**
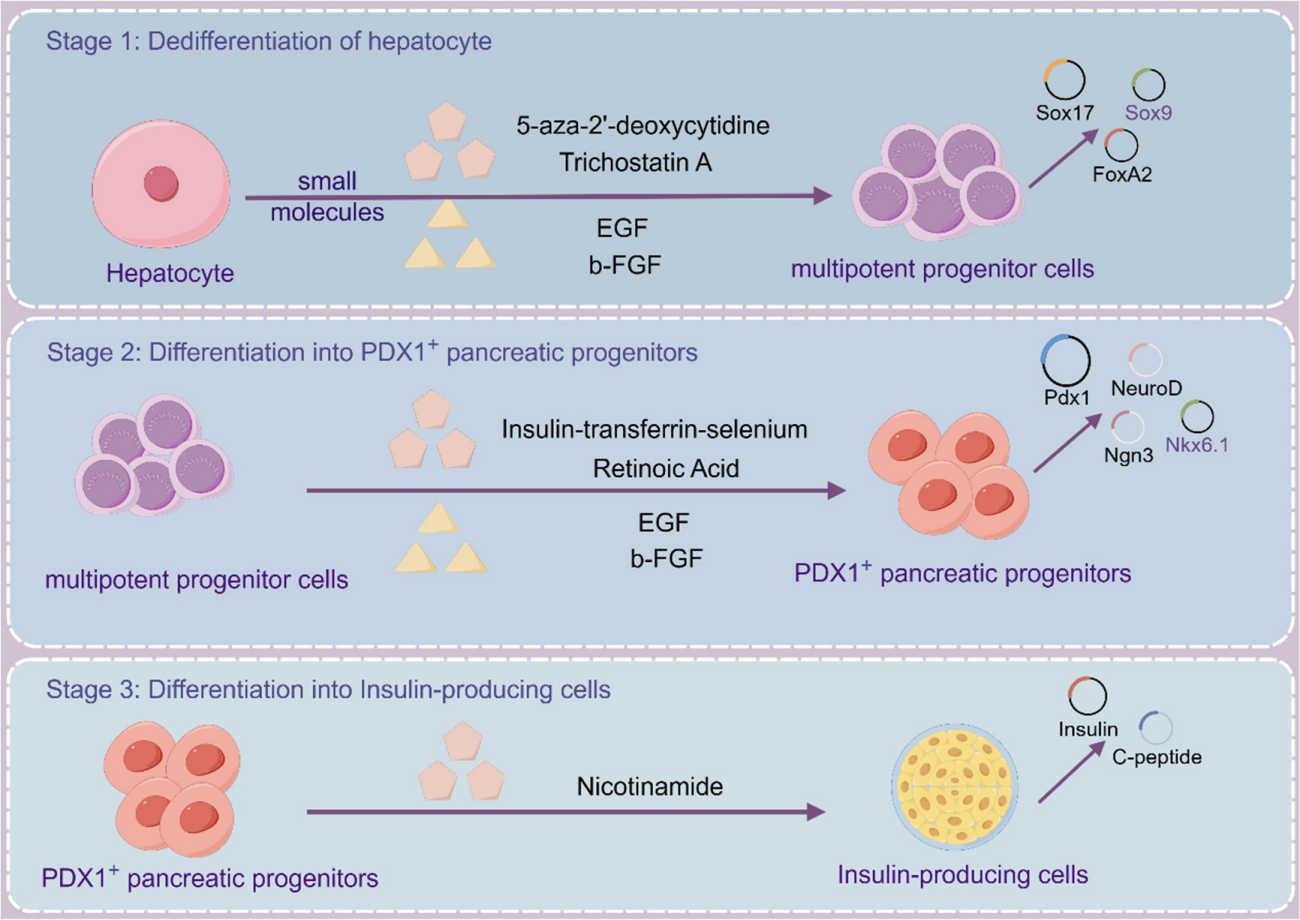
Induction of insulin‐producing cells derived from hepatocytes using small molecules. Stage 1: dedifferentiation of hepatocytes into multipotent progenitor cells. Stage 2: differentiation of multipotent progenitor cells into PDX1^+^ pancreatic progenitors. Stage 3: differentiation of PDX1^+^ pancreatic progenitors into insulin‐producing cells. b‐FGF, basic‐fibroblast growth factor; EGF, epidermal growth factor; FoxA2, forkhead box protein A2; Ngn3, neurogenin 3; PDX1, pancreatic duodenal homeobox‐1; Sox, SRY‐box transcription factor.

Chemical reprogramming is a new strategy for cell transdifferentiation (Figure 3). Of note, a growing body of research suggests that the use of small‐molecule compounds has good feasibility and advantages in inducing cell fate switching.^40^ Small‐molecule can effectively regulate cell fate, with cells responding rapidly and reversibly to their effects. These compounds can be optimized through synthetic chemistry for enhanced effects. In 2006, D'Amour et al differentiated human embryonic stem cells (hESCs) into endocrine cells with the ability to secrete pancreatic hormones through a five‐step strategy of chemical reprogramming.^41^ During the first stage of differentiation, researchers used a combination of activin A and Wnt3a to differentiate hESCs into a Definitive endoderm (DE), a stage in which cells express the DE markers *Sox17* and *FOXA2*. In the second stage, activin A was removed, fibroblast growth factor 10 (FGF10) and KAAD‐cyclopamine were added, and definitive endoderm was induced into intestinal endoderm. The expression levels of endoderm marker proteins hepatocyte nuclear factor 1β (HNF1B) and HNF4A were increased, and the expression of the DE markers *CER* and C‐X‐C chemokine receptor type 4 (*CXCR4*) were downregulated. *Sox17* has consistently been expressed in HNF1B positive cells. In the third stage, intestinal endoderm cells were cultured in retinoic acid (RA), KAAD‐cyclopamine, and FGF10 medium, and the cells rapidly expressed *PDX1* and *HNF6*, and *HNF1B* and *HNF4A* simultaneously. In the fourth stage, PDX1‐positive intestinal endoderm cells were transformed into pancreatic and endocrine cells by DAPT and exendin‐4. In the fifth stage, the endocrine cells expressed insulin, glucagon, somatostatin, pancreatic polypeptide, and ghrelin after 15 days of induced differentiation by exendin‐4, insulin‐like growth factor 1 (IGF1), and hepatocyte growth factor. However, cells that differentiate after a series of small‐molecule treatments are not mature or truly human beta cells. Timothy J Kieffer and Douglas A. Researchers from both teams, referring to the aforementioned differentiation pathways, successfully induced human pluripotent stem cells to differentiate into mature functional human pancreatic β cells in vitro, respectively.^42^, ^43^

Hepatocytes as the main seed cells for islet β‐cell induced differentiation, compared with iPSCs and ESCs, hepatocytes have attracted attention because of more stable genomes, better histocompatibility, and safer clinical treatment. In 2013, Liu et al successfully induced rat hepatic epithelioid stem cells WB‐F344 into insulin‐secreting cells after three steps using chemical reprogramming (Figure 1).^44^ In the first stage, cells are under the action of 5‐azacitidine and trichostatin A, and initially small polygonal WB‐F344 cells change to spindle shape with proliferation. After stage 1, alpha‐fetoprotein and albumin were undetectable, indicating successful dedifferentiation. In the second phase, induce with insulin‐transferrin‐selenium and RA‐containing medium for 7 days. 5% of cells show activation of *PDX1* gene expression, along with *Ngn3*, *Nkx2.2*, and *INS1* genes associated with pancreatic development and β cell function. WB‐F344 cells weakly expressed *GLUT2*, *PC1/3*, and *PC2* and did not express the late pancreatic development genes *Pax4*, *Pax6*, and *MafA*. Efficient conversion of WB cells into PDX1‐expressing progenitor cells. WB cells from stage two were transferred to a nicotinamide‐containing medium for insulin‐producing cell differentiation. In the third stage, islet cell‐specific markers like *PDX1*, *NeuroD*, and *INS1* were expresses, along with *Pax4*, *Pax6*, *MafA*, *GK*, *Kir6.2*, and *INS2*. PDX1‐positive cells made up 10% and glucose stimulation showed increased insulin levels, indicating successful differentiation through chemical reprogramming.

## 4 ORGANOID TECHNOLOGY OF HEPATOCYTES IN PANCREATIC REGENERATIVE MEDICINE

**FIGURE 4:**
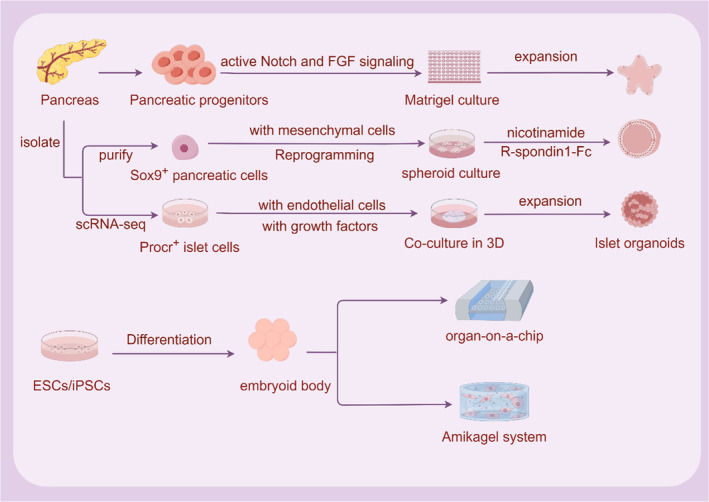
Organoid technology in pancreatic regenerative medicine. ESC, embryonic stem cell; FGF, fibroblast growth factor; iPSC, induced pluripotent stem cell; Sox9, SRY‐box transcription factor 9.

Cell dissociation and reaggregation experiments in classical developmental biology in the 20th century opened up the early exploration of in vitro mimicking organogenesis. In 1907, Wilson discovered that after mechanical dissociation, sponge cells could reaggregate themselves outside the body to become a new organism. Subsequently, in amphibians and chicken embryos, it was demonstrated that the dissociated cells had the ability to self‐organize. With the establishment of ESCs and iPSCs in in vitro culture systems, researchers have found that ESCs and iPSCs can form teratomas in vivo and embryoid bodies in vitro.^45^, ^46^, ^47^ ESCs and iPSCs not only have the ability to self‐renew and differentiate but also to self‐assemble into complex functional tissues and organs. In‐depth research on the extracellular matrix (ECM) has led to key breakthroughs in organoid culture (Figure 4). The construction of organoids is based on the self‐renewal, multi‐directional differentiation and self‐organization ability of stem cells, simulating the development path in vivo and differentiating into tiny organs of specific lineages. Based on ESCs, iPSCs, and adult stem cells, it provides a three‐dimensional (3D) culture microenvironment for cells, adds various induced differentiation factors in turn, artificially manipulates stem cell differentiation signaling pathways, enables stem cells to differentiate and self‐assemble according to the established pathway, and simulates the development process in vitro culture to generate organoid structures of multiple lineages or corresponding lineages.^48^, ^49^

Organoids, on the other hand, can best reflect human organs in vivo, bridging the gap between biological models at the cellular and tissue/organ levels.^50^ Using organoid technology, cells can self‐organize into organoids in vitro, self‐renewing while maintaining the promise of their native tissue, genetic stability, and potential to differentiate into functional cells in vitro (hepatocytes) and in vivo (hepatocytes and endocrine cells).^51^ Organoids are miniature organs with self‐renewal and self‐organization ability in vitro, which have a similar spatial organization to real organs and can perform primitive organ functions.

The pancreas is an organ with both endocrine and exocrine functions. The exocrine tissue of the pancreas is mainly composed of the acinar that secretes pancreatic juice and the pancreatic duct that drains pancreatic juice into the duodenum. Endocrine tissue consists of islets distributed between exocrine tissues. Pancreatic islets are heterogeneous structures formed by α, β, δ, and PP cells that secrete insulin, glucagon, somatostatin, and pancreatic polypeptide, respectively. Human islet α, β, δ, and PP cells are scattered in the islets, and β cells account for about 55% of the total number of islet cells.^52^, ^53^ Islet organoids are formed by embryo‐derived pancreatic cells, stem/progenitor cells of pancreatic islets, or stem cells of other sources inducing differentiation in vitro by 3D culture, and are similar to the 3D structure of islet composition and function in vivo. At present, islet organoids are mainly cultured in vitro from primary pancreatic tissue‐derived islet cells, hESCs, human iPSCs (hiPSCs), and so forth, and damaged pancreatic tissue is replaced by organ transplantation.^54^, ^55^

### 4.1 Islet organoids based on pancreatic cell origin

By day 11.5 of the mouse embryonic stage, the pancreas is essentially made up of pluripotent pancreatic progenitor cells that can differentiate into all types of pancreatic cells. These cells express *Sox9*, *PDX1*, *Hnf1b*, and *Ptf1a*.^24^, ^56^ In 2013, Greggio et al isolated embryonic pancreatic dorsal from mouse E10.5 to prepare pancreatic progenitor cell suspension and established a pancreatic progenitor cell culture medium with DMEM/F12‐based medium and the addition of serum‐free substitutes and β‐mercaptoethanol, phorbol myristate acetate, Y‐27632, epidermal growth factor (EGF), mouse R‐spondin1, FGF10, FGF1, heparin, and other small molecule progenitor cell culture media.^57^ The cell suspension was mixed with growth factor‐removed Matrigel and cultured in a 96‐well plate. In this 3D culture system, the dissociated mouse embryonic pancreatic progenitor cells were efficiently expanded. By adjusting the composition of the medium, hollow spheres consisting mainly of pancreatic progenitor cells can be generated, as well as complex organoids that spontaneously undergo pancreatic morphogenesis and differentiation. Experiments revealed the community effect of pancreatic development, through which small cell populations maintain the characteristics of progenitor cells better than isolated cells, as well as the requirement for 3D culture. Sox9^+^ pancreatic epithelial cells are considered pancreatic progenitor cells and are capable of developing into exocrine duct or acinar cells in vivo, as well as endocrine progenitor cells that produce all islet cells.^56^, ^58^, ^59^

Sugiyama et al isolated and purified single Sox9^+^ pancreatic epithelial cells from mouse embryo E11.5, similarly mixed early Sox9^+^ pancreatic epithelial cells with growth factor‐removed Matrigel and cultured under the condition of supplementation with insulin, transferrin, and IGF1 in PrEBM‐based medium. To achieve cell expansion and growth spheroids, cells were cultured at 21% oxygen concentration and supplemented with 150 ng/mL human FGF10, 2% (vol/vol) B27, and 0.33 μM all‐trans retinoic acid in basal medium. To induce spheroid differentiation, spheroids were cultured at 5% oxygen concentration and supplemented with basal medium with 50 ng/mL FGF10, 10 mM nicotinamide. The resulting islet organoids were cultured at 21% oxygen. Basal medium was supplemented with 150 ng/mL FGF10, 10 mM nicotinamide, and 0.5 mg/mL mouse R‐spondin1‐Fc or 200 ng/mL purified Wnt3a. By establishing an in vitro culture system, islet organoids with self‐proliferation and multidirectional differentiation potential were successfully obtained. Mouse embryonic pancreatic progenitor cells cultured under 3D conditions reflect in vitro differentiation of exocrine and endocrine cells but do not support the long‐term expansion of pancreatic cells. Tissue culture strategies using adult ducts, acinar, and low insulin cells have only limited function for expanding and differentiating presumed progenitor cells as β cells.^60^

In 2022, Wang et al identified a population of protein C receptor positive (Procr^+^) cells from the adult mouse pancreas by single‐cell RNA sequencing (scRNA‐seq).^61^ These cells are located in islets, do not express differentiation markers, and are characterized by epithelial‐mesenchymal transformation. Through genetic lineage tracing, Procr^+^ islet cells undergo clonal expansion and produce all four endocrine cell types during adult homeostasis. Sorted Procr^+^ cells make up approximately 1% of islet cells, and when cultured at clonal density, islet‐like organoids can be formed. β cells dominate in differentiated islet organoids, whereas α, δ, and PP cells occur less frequently. Organoids respond to glucose and secrete insulin. After transplantation in diabetic mice, these organoids can reverse STZ‐induced diabetes in mice.^62^

### 4.2 Islet organoids based on ESCs and iPSCs sources

In the past few decades, the in‐depth study of ESCs and iPSCs to islet β cell differentiation has provided a solid foundation for ESCs and iPSCs to islet organoid differentiation. In 2016, Kim et al constructed islet‐like clusters derived from hESCs and secreted glucose‐responsive insulin, which lowered blood sugar after the transplantation of STZ‐induced diabetic mice.^63^ In 2017, Chaimov et al developed a novel artificial pancreas encapsulation platform based on solubilized whole porcine pancreatic ECM. The ECM‐microcapsule platform provides a natural fibrous 3D niche that differentiates captured hepatocytes into insulin‐producing cells through ectopic expression of PDX1, Pax4, and MafA. In vivo, ECM‐encapsulated cells have been shown to be nonimmunogenic and, most important, can significantly improve glycemic control in preclinical models of diabetic mice.^64^ In 2018, Candiello et al developed a novel class of hydrogel systems, Amikagel, that generate regenerative islet organoids with precise size and cellular heterogeneity on this gel. Amikagel‐induced hESC‐PP spheroid formation enhances the expression of the β cell‐specific *INS1* gene and C‐peptide protein, as well as functional insulin production in response to glucose stimulation in vitro.^65^ In 2019, Tao et al used an organ‐on‐a‐chip platform to induce the differentiation of iPSCs into islet organoids. The organ microarray platform contains a multilayer microfluidic device that enhances the expression of pancreatic β‐cell‐specific genes *PDX1* and *Nkx6.1* under perfusion 3D culture conditions compared with static culture, at the same time promoting the increase of insulin secretion.^66^ Hepatocytes present themselves as favorable candidates for reprogramming into differentiated insulin‐producing cells (DIPCs) due to their shared developmental lineage with pancreatic cells, alongside their superior safety profile in comparison to stem cells (Table 1). In 2020, Lee et al fabricated DIPC spheroids derived from human hepatocytes using concave microwells. Higher levels of islet‐related gene expression and insulin secretion were observed in spheroids compared to single‐cell DIPC.^67^ This suggests that the attainment of a uniform size for the spheroids could potentially enhance the functionality of DIPC.

**TABLE 1:** Organoid technology of hepatocytes in pancreatic regenerative medicine.

Published year	Cell resource	Materials	3D structure	References
2013	Primary pancreatic islet cells and hepatocytes	Polydimethylsiloxane	Concave microwell arrays	68
2014	PP digested from human fetal pancreas and liver stromal cells derived from human fetal liver	‐	Self‐clustering in ultra‐low attachment plates	69
2017	Human liver cells and mesenchymal stem cells	Porcine pancreatic ECM	ECM‐microcapsule platform	64
2019	Human liver stem‐like cells forming 3D structure, followed with islet differentiation	‐	Based on charge dependent aggregation of HLSC induced by protamine	70
2020	Transdifferentiation of liver cells into DIPCs	Concave microwells	DIPC spheroid	67
2021	Insulin‐producing cells trans‐differentiated from human liver cells	poly(N‐isopropylacrylamide)	Engineered cell sheet	71
2022	Human induced pluripotent stem cell‐derived liver and islet organoids	Organoid coculture chip, peristaltic pump, and perfusion device	Microfluidic multiorganoid system	72

Abbreviations: 3D, three‐dimensional; DIPC, differentiated insulin‐producing cell; ECM, extracellular matrix; HLSC, human liver stem‐like cell; PP, pancreatic polypeptide.

### 4.3 Islet organoids based on coculture of islet cells with cells from other sources

With the maturation of in vitro induction of stem cell differentiation into islet‐like organs, how to avoid immune rejection and promote angiogenesis after islet‐like organ transplantation has become a problem that needs to be solved. As early as 2011, Jung et al cocultured bone marrow mesenchymal stem cells (BM‐MSCs) with isolated pancreatic islets and found that BM‐MSCs had a protective effect on cocultured and isolated pancreatic islet cells. After transplantation, pancreatic islets cocultured with BM‐MSCs showed stronger activity and glucose‐induced insulin secretion function. The levels of tissue inhibitor of metalloproteinase‐1 and vascular EGF increased at 4 weeks.^73^ In 2013, Jun et al used the concave micropores of polydimethylsiloxane to carry out 3D cocultures of primary islet cells and hepatocytes. They found that compared with the single cultured spheres, the islet spheres mixed with hepatocytes have higher stability and closer cell–cell junction, which improves the survival time of islet cell transplantation in vivo.^68^ In 2012, Kang et al transplanted endothelial progenitor cells from umbilical veins and porcine islets into nude mice with diabetes. The cotransplanted cells accelerated the vascular reconstruction of the graft, thus improving the functional state of the graft vascular system and greatly improving the transplantation β cell quality.^74^ In 2019, Lebreton et al successfully cocultured human amniotic epithelial cells (hAECs) with mouse pancreatic islet cells to produce a dynamic and functional pancreatic organ composed of hAECs and dissociated islet cells.^75^

### 4.4 Optimization of pancreatic islet organoid 3D culture protocol

Traditional cell differentiation research is mainly based on two‐dimensional (2D) cell culture, but 2D cell culture cannot simulate the complex spatial organization structure and complex physiological processes of cells in vivo.^48^, ^76^, ^77^ This article has reviewed the transformation of cells into pancreatic islet β‐like cells under a 2D culture system through various in vitro pathways, but the induced cells require specific tissue forms to perform normal physiological functions. Differentiated cells for regenerative applications, when using the established protocol, insulin‐expressing β‐like cells have low differentiation efficiency and final yield, lack functional characteristics, and show a lower ability to reverse disease conditions. In recent years, with the development of 3D culture technology, new ideas have been brought to the research and disease treatment of islet regenerative medicine. The natural ECM extracted from mouse sarcomas by Orkin et al provides a 3D environment for organoid culture. This ECM gel‐like protein derived from tumor cell lines has been shown to support the self‐renewal potential of stem cells and maintain their undifferentiated state.^78^ However, the heterogeneous cancer cell line origin of Matrigel hinders its medical use, as well as batch‐to‐batch differences, hindering the reproducibility of the study, resulting in the inability of islet organoids to be produced under Good Manufacturing Practice (GMP) conditions for clinical treatment. The use of chemically synthesized matrices for organoid culture has been well studied.

Currently, polyethylene glycol (PEG), polylactic acid, poly ε‐caprolactone, polyvinyl alcohol, and polylactide‐co‐glycolide artificial matrix gums synthesized from various materials are used to culture organoids.^79^, ^80^ Due to the lack of growth factors in synthetic Matrigels, the organoid field is pushed toward GMP by adding growth factors to specific media.^81^ Greggio et al crosslinked PEG hydrogels with laminin and thrombin activator XIIIa instead of Matrigel as a medium for pancreatic organoids. However, this hydrogel is less effective at maintaining the morphology and function of pancreatic embryonic organoids.^57^ Youngblood et al synthesized a hydrogel with micropores of 250 ~ 400 μm to make the organoids cultured in it have a controllable cell density and volume/area ratio. However, it has been suggested that support mediators affect the maturation status of pancreatic organoids.^82^ Candiello et al synthesized a novel hydrogel Amikagel and cultured pancreatic progenitor cells induced from hESCs on Amikagel and found that the cells could spontaneously aggregate into circular colonies and gradually transform into pancreatic organoids with the ECM. Compared with Matrigel, the pancreatic organoids obtained from Amikagel culture have a higher maturity and can respond better to sugar stimuli.^65^

The medium for pancreatic organoids was developed from the basis of intestinal organoids and consists of DMEM/F12 medium and EGF, Noggin, and R‐Spondin1.^83^ EGF promotes pancreatic cells while inhibiting the differentiation of pancreatic endocrine progenitor cells.^84^ Noggin, as a member of the transforming growth factor superfamily, is the basic cytokine of pancreatic development and the basis for the development of solid organs, including the pancreas.^85^ R‐Spondin1 is an agonist of the LGR5 receptor of the Wnt/β‐catenin pathway, which in turn is essential for the development and self‐renewal of several types of adult stem cells.^86^ At the same time, FGF10, nicotinamide, N‐acetylcysteine, and B27 are also widely used as components of pancreatic islet organoid media.^87^ FGF10 promotes PDX1^+^ pancreatic progenitor cell expansion and aids pancreatic progenitor cell growth and differentiation.^88^, ^89^ Nicotinamide induces fetal pancreatic endocrine cell differentiation and maturation and is commonly used for islet organoid culture.^90^, ^91^, ^92^ Pancreatic organoids are expected to be a new diabetes treatment, but further studies are needed to evaluate their safety and efficacy.

### 4.5 CRISPR/Cas9 gene editing in organoid technology

In only a decade, CRISPR‐associated nucleases were discovered to be capable of genome editing.^93^, ^94^ Traditional and emerging therapeutic modalities have been reshaped by this revolution. The ability of Cas9 to (a) identify nucleic acid sequences with high affinity and (b) precisely cleave target genes to trigger repair mechanisms to insert desired sequences has led to the development of several biological tools and translation applications.^95^, ^96^ Cho et al designed a nanocarrier consisting mainly of lecithin and self‐assembling Cas9 complexes with single‐stranded guide RNA (sgRNA) ribonucleoprotein (Cas9‐RNP) for injection into type 2 diabetes mellitus (T2DM) db/db mice.^97^ Cas9‐RNP complexes are efficiently delivered to hepatocytes through accumulation in the liver, disrupting the expression of the dipeptidyl peptidase 4 gene in T2DM mice while normalizing blood glucose levels and reducing liver and kidney damage. Studies have shown that liver‐specific transcriptional enhancers regulated by FOXA2 are present at the intron T2DM sites represented by rs780094, rs780095, and rs780096SNP, and the expression of glucokinase regulatory protein gene can be increased by using the CRISPR‐dCas9 transcriptional activator system.^98^ Future CRISPR‐Cas applications could design ways for hepatocytes to become β cells to circumvent the inherent limitations of β cells, including insufficient response to glucose‐stimulated insulin and susceptibility to immune attacks after transplantation.

## 5 CONCLUSIONS AND PROSPECTS

The application of emerging strategies such as organoid technology, scRNA‐seq, and CRISPR/Cas9 gene editing technology has greatly promoted the research progress of hepatocytes in pancreatic regenerative medicine. Hepatocytes have great potential in the field of pancreatic regenerative medicine; however, the validation of their safety and efficacy still needs to be strengthened. Compared with natural islets, organoids are generally immature and heterogeneous in terms of cell composition. Future research should focus on time‐specific signal transduction and reconstruction of the pancreatic endocrine microenvironment.

By integrating bioinformatics and genetic engineering techniques, the mechanism of hepatocytes in the process of pancreatic regeneration can be explored in order to find the most ideal cell regeneration strategy.

## AUTHOR CONTRIBUTIONS

Shuang Liu and JiainPing Liu designed this study. Shuang Liu performed the literature review and wrote the manuscript. YuYing Zhang and YunFei Luo helped with the review and writing of the manuscript. All authors read and approved the final manuscript.

## FUNDING INFORMATION

This work was supported by the National Natural Science Foundation of China (82160162 and 81760150), and the Natural Science Foundation of Jiangxi Provincial (20202ACBL206008).

## DISCLOSURE

The authors declare that they have no competing interests.
